# Comparison of LC-PUFAs Biosynthetic Characteristics in Male and Female Tilapia at Different Ontogenetic Stages

**DOI:** 10.3390/life15081167

**Published:** 2025-07-23

**Authors:** Fang Chen, Liuling Gao, Junfeng Guan, Chao Xu, Deshou Wang, Yuanyou Li, Dizhi Xie

**Affiliations:** 1 College of Marine Sciences, South China Agricultural University, Guangzhou 510642, China; 2 Institute of Eco-Environmental Research, Guangxi Academy of Sciences, Nanning 530007, China; 3 School of Life Science, Southwest University, Chongqing 400715, China

**Keywords:** tilapia, LC-PUFA biosynthesis capacity, ontogenetic processes, fatty acid compositions

## Abstract

Tilapia possess the ability to biosynthesize long-chain polyunsaturated fatty acids (LC-PUFA); however, variations in this capacity across different growth stages and between sexes remain poorly understood. This study evaluated the LC-PUFA biosynthetic capacity in male and female tilapia fed two distinct diets—perilla oil (rich in α-linolenic acid, ALA) and peanut oil (rich in linoleic acid, LA)—over 24 weeks, spanning four growth stages (I-IV, from fry to adult). The results revealed that during stages I to III, both diets produced similar final body weights. However, in stage IV, male tilapia fed the peanut oil diet exhibited significantly higher body weight compared to those fed perilla oil, whereas females showed no significant differences between diets. Throughout stages III and IV, males were consistently heavier than females. LC-PUFA levels in the liver and intestine varied across growth stages, with the lowest levels at stage II and the highest at stage III. Notably, male tilapia exhibited higher expression levels of *fads2* and *elovl5* compared to the females across stages II to IV. The hepatic and intestinal mRNA levels increased by up to 6.40-fold and 3.85-fold, respectively, indicating a greater LC-PUFA biosynthetic capacity in males. This study provides valuable insights into the biosynthesis of LC-PUFA in tilapia, highlighting the influence of growth stage, sex and dietary fatty acid composition on this process, and laying a foundation for further evaluating the functional significance of dietary lipid sources in aquaculture.

## 1. Introduction

Long-chain polyunsaturated fatty acids (LC-PUFAs), particularly arachidonic (ARA, 20:4n-6), eicosapentaenoic (EPA, 20:5n-3) and docosahexaenoic acid (DHA, 22:6n-3), are essential fatty acids (EFAs) indispensable for the survival, health, growth and reproductive performance of fish [1,2]. In aquatic feeds, natural LC-PUFA sources primarily derive from marine products, especially fish meals and fish oil. However, the rapid expansion of aquaculture has exacerbated the persistent supply shortages of these marine-derived resources [3]. To reduce reliance on marine inputs, plant-based sources, rich in n-6 PUFA but lacking LC-PUFA, have been extensively utilized in aquatic feeds [4]. This substitution often results in EFA deficiencies, leading to metabolic disorders, impaired immune responses, and diminished nutritional quality of farmed fish [5,6,7]. Research on LC-PUFA requirements in teleosts indicates that the physiological needs for survival are relatively low. However, higher LC-PUFA levels are critical for optimal growth and health, particularly in juveniles, with increased requirements during early developmental stages (larvae) and reproductive phases [3]. These findings underscore that the nutritional demands for LC-PUFA vary across developmental stages, emphasizing the importance of understanding the precise EFA requirements to promote optimal growth and reduce reliance on marine ingredients in aquafeeds [3,4].

Historically, it has been widely acknowledged that freshwater and migratory fish species can convert linoleic acid (LA, 18:2n-6) and α-linolenic acid (ALA, 18:3n-3) into LC-PUFA, thereby fulfilling their EFA requirements through the dietary intake of LA and ALA present in vegetable oils (VOs). Conversely, most marine teleosts, with a limited capacity for LC-PUFA biosynthesis, rely on dietary sources of these essential biomolecules [4,8]. Furthermore, during the migratory phase, species capable of endogenous LC-PUFAs synthesis exhibit increased needs for these fatty acids to support rapid physiological adaptation to fluctuating environmental conditions [9]. As previously noted, the requirements for LC-PUFA vary among fish species and across developmental stages, indicating that biosynthetic capacity may also be modulated throughout growth. Although research on stage-specific LC-PUFA biosynthetic capacity remains limited, studies have reported notable changes in the expression of key enzymes involved in LC-PUFA biosynthesis during various stages of embryonic development, suggesting that the capacity to biosynthesize LC-PUFA is likely developmentally regulated in fish [10,11,12].

Tilapia, like other freshwater fish species, possesses a complete set of endogenous enzymes (∆6∆5 Fads2, ∆4 Fads2, Elovl5) that enable the biosynthesis of LC-PUFA from LA and ALA [13]. The efficiency of converting C18 PUFA to LC-PUFA in tilapia has been shown to be modulated by various external factors, including dietary fatty acid composition, environmental salinity and temperature [14,15,16,17,18]. While previous studies have explored the impact of dietary lipid sources and fatty acids on the growth, development, and reproductive performance of tilapia [19,20], information on the species’ LC-PUFA biosynthetic capacity across different growth stages remains limited. In this study, we evaluated the differences in LC-PUFA biosynthesis between male and female tilapia at the fry, juvenile, and adult stages. These findings provide new insights into the developmental regulation of LC-PUFA biosynthesis in fish and contribute to the formulation of nutritionally balanced diets that meet EFA requirements throughout different growth stages.

## 2. Materials and Methods

### 2.1. Experimental Diets and Fish

Two isolipidic (7.50%) and isoproteic (36.5%) diets were formulated, designated as PO and PT. The PO diet, using perilla oil as the lipid source, was rich in ALA at 3.86%, whereas the PT diet, formulated with peanut oil, had a much lower ALA content (0.07%). For tilapia fry, feed ingredients were sifted through a 120-mesh sieve and thoroughly blended with 25% water in an electric blender for 15 min. The mixture was then pelletized (Φ 1 mm) using a feed-pelletizer (Shanghai, China). After drying, the diets were crushed and passed through a 40-mesh sieve, and the final powdered feed was kept at −20 °C until use. For juvenile tilapia, all feed ingredients were sieved through a 60-mesh screen and similarly blended with 25% water for 15 min before being pelletized (Φ 1.5 mm). The diets were sealed in plastic bags and preserved at −20 °C after drying. Both diets contained high levels of C18 PUFAs but were free of LC-PUFAs. The detailed information on feed ingredients, proximate composition and fatty acid profiles is provided in Table 1.

Male (XY) and female (XX) tilapia fry were provided by Prof. Deshou Wang (School of Life Sciences, Southwest University) and maintained at the breeding facility of South China Agricultural University.

### 2.2. Fish Husbandry, Weighing and Sampling

Prior to the experiment, juvenile tilapia were acclimatized for one week and fed a commercial tilapia diet. Following a 24 h fasting period, 600 fish (300 females and 300 males) of similar size with an initial body weight of approximately 0.3 g, were randomly allocated to twelve tanks (Φ: 0.6 m, D: 1 m), with 50 fish per tank and triplicate tanks per dietary treatment. The feeding trial lasted for 24 weeks and was systematically divided into four sequential stages: stage I and stage II (each lasting 4 weeks), followed by stage III and stage IV (each lasting 8 weeks). During stage I, fish fry was fed to satiation five times a day (at 9:00, 11:00, 13:00, 15:00 and 17:00). In the subsequent stages (II–IV), feeding frequency was reduced to twice daily, at 9:00 and 17:00. Throughout the entire feeding trial, half of the water volume in each tank was exchanged daily at 18:00, and continuous aeration was maintained. The water temperature was kept at 25 ± 3 °C under natural light conditions.

At the start of the feeding trial, 10 whole fish were collected from both male and female tilapia, respectively. Fish were fasted for 24 h, anesthetized with phenoxyethanol, weighed and sampled at the end of each feeding stage. For stage IV, three fish from each tank were sampled for liver, intestine, muscle and gonads tissues. All samples were rapidly frozen in liquid nitrogen and preserved at −80 °C for subsequent analysis.

### 2.3. Analysis of Proximate and Fatty Acid Compositions

The proximate composition of the experimental diets was determined following the methods outlined in our previous study [16]. Briefly, crude ash content was measured by combusting the samples in a muffle furnace at 550 °C until a constant weight was achieved. Moisture content was determined by drying the samples at 80 °C to a constant weight. Total nitrogen (N) was analyzed using the Kjeldahl method after acid digestion, and the crude protein content was calculated as N % × 6.25. Crude lipid content was assessed using Soxhlet extraction. All measurements were conducted in triplicate.

Fatty acids from diets, whole body, intestine, liver, muscle and gonad were extracted following the method described by the authors of [16]. Briefly, the samples were homogenized and extracted with a chloroform/methanol mixture (*V*/*V*, 2:1) containing 0.01% butylated hydroxytoluene as an antioxidant. Fatty acid methyl esters (FAMEs) were then prepared via methyl esterification using boron trifluoride in diethyl etherate (Acros Organics, Waltham, MA, USA). The resulting FAMEs were dissolved in n-hexane and analyzed using a gas chromatograph (Agilent Technologies, Palo Alto, CA, USA). Individual fatty acids were identified by comparison with commercial FAME standards (Sigma-Aldrich, Saint Louis, MO, USA) and quantified through the Area Normalization method.

### 2.4. Quantitative Real-Time PCR (qRT-PCR)

To assess changes in LC-PUFA biosynthesis in tilapia across the four feeding stages, the relative mRNA expression levels of key enzyme genes involved in LC-PUFA biosynthesis (Δ4 fads2, Δ6Δ5 fads2 and elovl5) were measured in the liver and intestine. Total RNA was extracted from the liver and intestine using RNAiso Plus (Takara, Dalian, China) following the manufacturer’s instructions. The first-strand cDNA was synthesized from 1 μg of RNA using a commercial reverse transcription kit (Takara, Dalian, China), according to the provided protocol. Quantitative PCR (qPCR) was performed using gene-specific primers (Table 2). Each 10 μL qPCR reaction mixture consisted of 3.5 μL ddH_2_O, 5 μL SYBR Green I Master (TOYOBO, Tokyo, Japan), 0.5 μL of primer (10 μM) and 1 μL cDNA (50 ng/μL). Amplification was carried out on a Lightcycler 480 system (Roche, Basel, Switzerland), with each sample analyzed in triplicate. The relative mRNA expression levels were calculated using the 2^−ΔΔCT^ method, with β-actin serving as the internal control.

### 2.5. Histological Observation

For histological observation, the prefixed gonad samples from three fish per group were dehydrated in a graded ethanol series, embedded in paraffin, and then sectioned into 5 μm slices using a Leica RM 2035 microtome (Leica Microsystems Ltd., Milton Keynes, UK). The tissue sections were stained with haematoxylin-eosin and Alcian blue (8 GX, pH 2.5), and examined under a light microscopy (Olympus, CX21, Tokyo, Japan).

### 2.6. Statistical Analysis

All data are presented as mean ± standard error of the mean (SEM, *n* = 3). Paired *t*-test has been used to directly compare the body weight and tissue FA composition (%) between fish fed the PO or PT diets at the same feeding stage. Differences in the tissue FA composition (%) of fish fed the same diets across the four feeding stages were analyzed using one–way analysis of variance (ANOVA), followed by Turkey’s multiple comparison test. *p* < 0.05 was considered statistically significant. Heatmap analysis was conducted using Graphpad Prism 8.0 software to visualize the relationship between tissue FA composition and growth stages across different dietary groups. Additionally, principal component analysis (PCA) was performed using SIMCA 14.1 to identify variations in tissue LC-PUFA contents across the four growth stages.

## 3. Results

### 3.1. Final Weight of Fish Across Four Growth Stages

**Figure 1:**
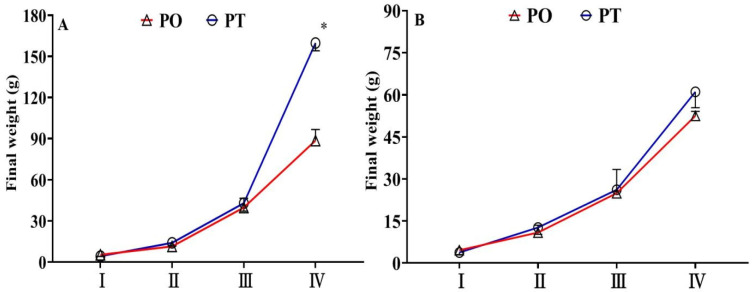
The final weight (g) of male (**A**) and female (**B**) tilapia during the four-growth stage. Values are means ± SEM (*n* = 3), and asterisk represents significant differences between PO and PT groups during the same feeding stage (*p* < 0.05). The detailed values were listed in Table S1.

The final weight of male and female tilapia fed the PO and PT diets across the four growth stages is shown in Figure 1 and Table S1. During stages I–III, the final weight of tilapia fed the PO diet was generally higher than those fed the PT diet; however, these differences were not statistically significant (*p* > 0.05). At stage IV, the final weight of female tilapia fed the PT diet was approximately 16% higher than those fed the PO diet. Notably, male tilapia fed the PT diet had significantly higher final weight compared to those fed the PO diet (*p* < 0.05). Interestingly, sex-based differences in growth were also evident. At stages III and IV, the final weight of male tilapia fed the PO diet was 59.81% and 68.75% higher, respectively, than their female counterparts. Similarly, male tilapia fed the PT diet had final weights that were 64.36% and 161.57% higher than those of females, respectively.

### 3.2. Initial Fatty Acid Compositions of Whole Fish

The fatty acid compositions of the initial whole fish are shown in Table 3. Saturated fatty acids (SFAs), monounsaturated fatty acids (MUFAs) and n-3 LC-PUFA are the predominant fatty acids in both male and female tilapia. In particular, the content of n-3 LC-PUFA (EPA, DPA and DHA) exceeded 20.0%, while the content of n-6 LC-PUFA remained below 3.7%. The fatty acid composition of male and female tilapia exhibited subtle differences. For example, male tilapia showed slightly higher levels of total n-3 PUFA, n-3 LC-PUFA and n-6 LC-PUFA compared to females, except for total n-6 PUFAs, which were higher in females. Additionally, females had a marginally higher proportion of MUFAs, while males exhibited a slightly higher SFA content.

### 3.3. Fatty Acid Composition in the Liver, Intestine and Muscle of Tilapia Across Four Stages

**Figure 2:**
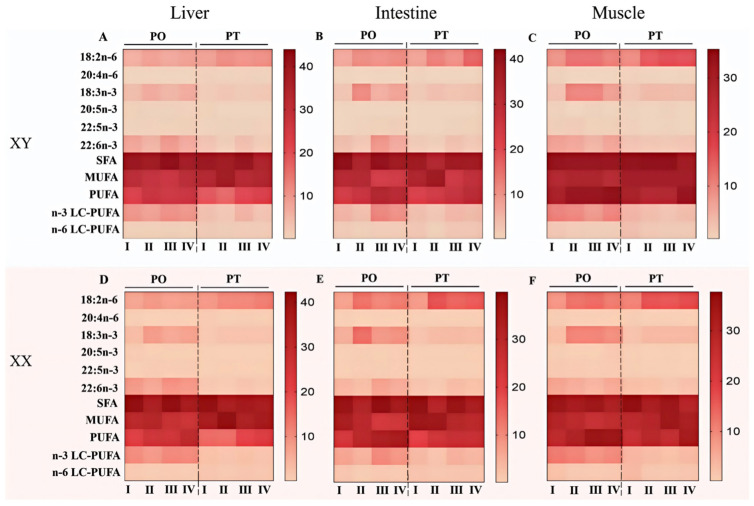
Main fatty acid contents in the liver (**A**,**D**), intestine (**B**,**E**) and muscle (**C**,**F**) of tilapia among the four-growth stages. Values are means ± SEM (*n* = 3). The detailed values were listed in Tables S2–S4.

The fatty acid profiles of the liver, intestine and muscle tissue of fish across different growth stages are shown in Figure 2 and Tables S3 and S4. Overall, the patterns of fatty acid composition were similar between female and male fish across different growth stages. At the same stage, fish fed the PO diet consistently exhibited significantly higher levels of n-3 fatty acids (ALA, EPA, and DHA) in all examined tissues compared to those fed the PT diet (*p* < 0.05). In contrast, fish fed the PT diet showed significantly higher levels of LA, ARA and n-6 LC-PUFA. Within both dietary groups, the tissue levels of LA and ALA at stage I were significantly lower than those at stages II–IV (*p* < 0.05).

Interestingly, the patterns of fatty acid composition varied distinctly across tissues during different growth stages. For instance, in the liver (Figure 2A,D, Table S3), the levels of both n-3 and n-6 LC-PUFA were significantly higher at stages I and III compared to stage II (*p* < 0.05). Additionally, the content of EPA was significantly higher at stage I relative to stages II–IV (*p* < 0.05), while the DPA level was comparatively lower (*p* > 0.05). The levels of n-3 LC-PUFA and n-6 LC-PUFA were relatively higher at stage III compared to stages II and IV, whereas the levels of LA and ALA tended to be lower. However, these differences did not reach statistical significance. Additionally, the liver fatty acid profiles reveal sex-specific differences in LC-PUFA dynamics across growth stages, modulated by diets (PO vs. PT). Females and males under PO diets tended to maintain similar total n-3 LC-PUFA and n-6 LC-PUFA contents. In contrast, males under PT diets exhibited superior contents of n-3 LC-PUFA, particularly in later stages (II and IV). Both sexes showed stage-dependent fluctuations, with stage II consistently associated with reduced LC-PUFA levels, possibly reflecting ontogenetic shifts in metabolic demands.

Regarding the intestinal fatty acid composition across different growth stages (Figure 2B,E, Table S3), the levels of n-3 LC-PUFA (EPA, DPA, DHA) in the PO groups were significantly higher at stages II and IV compared to stages I and II (*p* < 0.05). Additionally, n-6 LC-PUFA content was significantly lower at stage II compared to the other stages (*p* < 0.05). In the PT groups, both n-3 LC-PUFA (EPA, DPA, DHA) and n-6 LC-PUFA levels were significantly lower at stage II compared to the other stages (*p* < 0.05). Key LC-PUFA precursors (ALA and LA) peaked in stage II, reflecting the potential low LC-PUFA biosynthesis in this growth stage. When comparing stage IV and III, stage III exhibited higher levels of both n-3 and n-6 LC-PUFA. However, the levels of LA and ALA remained relatively low and did not differ significantly between these two growth stages. Sex differences were most pronounced under PO diets: males exhibited superior total n-3 LC-PUFA contents in stage III, driven by higher DHA, while females showed higher EPA contents in stage IV.

For the fatty acid composition of muscle across the four growth stages (Figure 2C,F, Table S4), the levels of n-3 LC-PUFA (EPA, DPA, DHA) were lower at stage III compared to the other stages. Stage I exhibited significantly higher levels of both n-6 and n-3 LC-PUFA than stages II–IV and III (*p* < 0.05), respectively. Within stages II–IV, stage III showed lower levels of both n-6 and n-3 LC-PUFA compared to stages II and IV (with a significant decrease observed in n-3 LC-PUFA), while exhibiting relatively higher levels of LA and ALA. Muscle fatty acid profiles highlight sex-specific adaptations: males fed with PO diets maintain high DHA and total n-3 LC-PUFA in the latest stage (IV) than their female counterparts.

### 3.4. Differentiation of Fish Across Different Growth Stages Based on the LC-PUFA Contents

**Figure 3:**
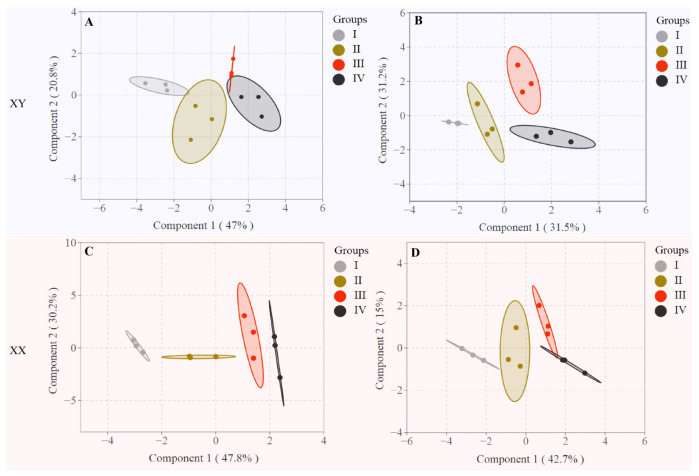
Principal component analysis of LC-PUFA contents in the liver, intestine and muscle of male and female tilapia fed with diets PO (**A**,**C**) and PT (**B**,**D**). The detailed values were listed in Tables S2–S4.

The PCA method was employed to better understand the relationship between tissue FA composition and growth stages. As depicted in Figure 3, the cumulative contribution of the first two principal components exceeded 60%, effectively capturing the essential characteristics of the original dataset. The PCA score plot based on LC-PUFA contents revealed clear stage-specific clustering patterns. Regardless of sex or dietary lipid source, samples from growth stages I and II formed a cohesive cluster, which was distinctly separated from those of stages III and IV. Sex-specific divergence is most pronounced in stage III under PO diets, reflecting tissue-specific differences in LC-PUFA contents (e.g., higher n-3 LC-PUFA in male intestine and liver). In contrast, PT diets reduce both stage and sex differentiation, likely due to lower overall LC-PUFA availability.

### 3.5. Expression of Genes Involved in LC-PUFA Biosynthesis in Fish at Four Different Growth Stages

**Figure 4:**
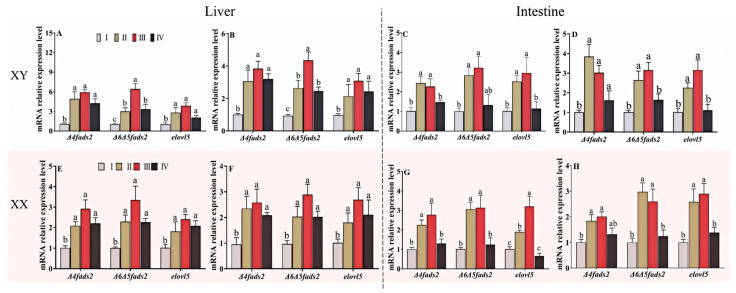
mRNA expression of Δ4 *fads2*, Δ6/Δ5 *fads2* and *elovl5* relative to *β-actin* in the male and female tilapia fed with diets PO (**A**,**C**,**E**,**G**) or PT (**B**,**D**,**F**,**H**) among the four-growth stages. Values are means ± SEM (*n* = 3), and different letters on bars represented significant differences among the four growth stages (*p* < 0.05).

Figure 4 illustrates the expression levels of key genes (Δ4 *fads2*, Δ6Δ5 *fads2*, *elovl5*) involved in the biosynthesis of LC-PUFA in the intestine and liver. The expression patterns of these genes were consistent across growth stages in both female and male fish, regardless of dietary lipid source. Hepatic expression levels of *fads2* and *elovl5* were significantly higher at stages II–IV compared to stage I (*p* < 0.05) (Figure 4A,B,E,F). Similarly, intestinal *fads2* and *elovl5* expression levels were elevated at stages II–III compared to those at stages I and IV (Figure 4C,D,G,H). Overall, peak expression of these biosynthetic genes in both liver and intestine predominantly occurred at stage III, although differences among stages II–IV were generally not statistically significant.

The mRNA expression patterns of key enzymatic genes reveal stage-specific sex divergence. Compared to stage I, the hepatic *fads2* and *elovl5* expression levels in male tilapia increased by 2.45 to 6.40 fold and 2.05 to 3.85 fold, respectively, during stages II–IV. For female fish, the corresponding increases were 2.02 to 3.34 fold for *fads2* and 1.80 to 2.69 fold for *elovl5*. At stages II and III, the expression of intestinal *fads2* and *elovl5* in males increased by 2.46 to 3.84 fold and 2.25 to 3.15 fold, respectively, while in females, the increase ranged from 1.81 to 3.12 fold for *fads2* and 1.90–3.19 fold for *elovl5*.

### 3.6. Gonad Fatty Acid Compositions and Morphology at Stage IV

The gonad fatty acid compositions of tilapia at stage IV are shown in Table 4. Fish fed the PO diet exhibited significantly higher levels of n-3 LC-PUFA (including 20:3n-3, EPA, DPA, DHA) in both the testes and ovaries, compared to those fed the PT diet (*p* < 0.05), while the n-6 LC-PUFA content (ARA) exhibited an opposite trend (*p* < 0.05). Interestingly, within each dietary group, the testes contained higher levels of ARA and other n-6 LC-PUFAs than those of the ovaries.

**Figure 5:**
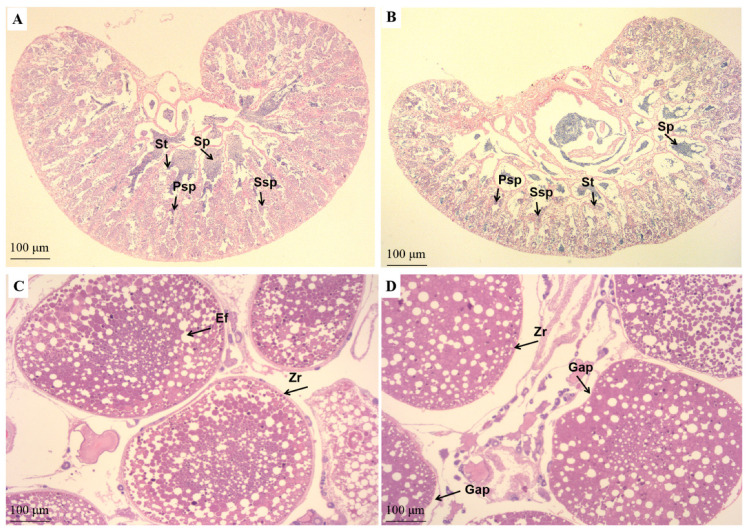
Histological section analysis of the testes (**A**,**B**) and ovaries (**C**,**D**) of adult tilapia fed with diets PO (**A**,**C**) or PT (**B**,**D**) during the fourth growth period. Psp, primary spermatocytes; Ssp, secondary spermatocytes; St, spermatids; Sp, spermatozoa; Ef, empty follicle; ZR. zona radiata.

The histological characteristics of the testes and ovaries are presented in Figure 5 and Figure S1. The testes from fish fed either the PO or PT diets exhibited multi-stage spermatogenesis, characterized by the presence of larger spermatogonia or spermatocytes along with numerous smaller sperm cells. Notably, in comparison to the PO group (Figure 5A and Figure S1A), the PT group (Figure 5B and Figure S1B) displayed a greater abundance of sperm cells and a more organized cellular structure, indicating superior testicular development. In contrast, the development of ovaries displayed comparable characteristics between the PO and PT groups (Figure 5C,D and Figure S1C,D). In both groups, mature oocytes were observed with yolk granules fully occupying the cytoplasm, which were stained reddish orange. These granules had fused to form yolk plates. The nuclear membrane had dissolved, and the follicular membrane had ruptured, allowing for the release of mature oocytes, leaving the empty follicular structures behind. However, subtle differences were noted in the PT group (Figure 5D and Figure S1D), where the zona radiata appeared thinner, and a distinct separation between the zona radiata and the chorion was observed. This structural difference may have facilitated the detachment of the mature oocyte from the surrounding tissue, in contrast to the PO group (Figure 5C and Figure S1C).

## 4. Discussion

### 4.1. EFA Requirements of Tilapia Vary at Different Growth Stages

The essential roles of n-3 LC-PUFAs, such as EPA and DHA, in fish growth and development have been widely recognized [1,2]. Previous studies have reported that whole-body EPA and DHA contents in tilapia fry are consistently around 15% [21]. In line with these findings, our study observed initial EPA and DHA levels of approximately 20% in tilapia fry, further emphasizing the critical importance of n-3 LC-PUFAs during early development. As a freshwater species, tilapia can endogenously convert ALA into n-3 LC-PUFAs, making dietary ALA an essential precursor for their growth and survival. However, the preliminary studies indicated that a dietary supplement of approximately 1.0% LA is sufficient to meet the EFA requirement of juvenile tilapia [22,23]. Interestingly, in the present study, no significant difference in growth was observed between tilapia fry fed a low ALA diet (PT, 0.07% ALA) and those fed a high ALA diet (PO, 3.86% ALA) during stage I (0.4–4.5 g). These seemingly contradictory results may be explained by the following two key factors. First, as functional fatty acids, LC-PUFAs are accumulated in tissue structures, and, thus, early nutritional history can influence the outcomes of subsequent growth performance [24]. Second, tilapia possess a robust capacity to biosynthesize n-3 LC-PUFA from limited dietary ALA, which may be sufficient to meet their physiological requirements [16]. The comparable growth performance observed between the PO and PT groups during stages II and III further supports the hypothesis that tilapia can compensate for lower dietary ALA through efficient endogenous conversion.

Meanwhile, at stage IV, male tilapia in the PT group showed significantly improved growth performance compared to those in the PO group. Although no statistically significant difference in growth was observed between the two female groups, the final weight of females in the PT group was 16.28% higher than that in the PO group. Histological analysis of gonadal tissue further indicated that the PT diet, rich in LA, was more beneficial for gonadal development and gametogenesis. These results suggest that higher levels of dietary n-6 fatty acid (LA) are required during the gonadal maturation phase in tilapia. This finding is consistent with previous studies reporting significant improvements in egg quality and offspring performance following ARA supplementation in cultured broodstock [25,26,27,28]. Similarly, numerous studies have shown that both larvae and broodstock require higher levels of EFAs than juveniles and sub-adult fish to meet the metabolic demands for rapid growth and reproduction [29]. These findings suggest that EFA requirements vary across developmental stages, reflecting the shifting physiological priorities of the fish. Therefore, for fish species with LC-PUFA biosynthesis ability, both the quantitative requirement for EFAs and the regulation of LC-PUFA metabolic pathways are likely modulated in a stage-specific manner through development.

### 4.2. Variations in the Biosynthetic Capacity of LC-PUFA in Tilapia at Different Growth Stages

The fatty acid composition of fish tissues is affected by both dietary fatty acid profiles and endogenous metabolic processes, as is the case with other nutrients [30,31]. Numerous in vitro and in vivo studies have demonstrated that the liver and intestine are the primary sites for LC-PUFA biosynthesis in fish, particularly in freshwater species [16,32,33]. The levels of LC-PUFA, as well as their precursors and intermediate metabolites in the liver and intestine, varied across developmental stages, reflecting ontogenetic shifts in the biosynthetic capacity for LC-PUFA [16,31]. At stage I (fry to early juveniles), tilapia exhibited significantly lower levels of DHA and ARA in the liver and intestine compared to stage II (juveniles), which showed higher levels of metabolic intermediates such as 20:3n6 and 22:5n3. The high retention of LC-PUFA in fry tissues during early development suggests a limited endogenous capacity for LC-PUFA biosynthesis at this stage. This limitation may be attributed to the relatively high initial LC-PUFA levels (25%) in fry, which could suppress the expression of key biosynthetic enzyme genes (*fads2*, *elovl5*) involved in LC-PUFA biosynthesis. Similar findings have been reported in fish embryos, where developmental progression is accompanied by changes in LC-PUFA levels and their substrates, alongside stage-dependent modulation of *fads2* and *elovl5* [10,34,35,36]. In wild fish, dietary shifts associated with ontogeny further influence fatty acid metabolism and tissue LC-PUFA content [37]. Consistent with these observations, our study revealed that tilapia at stage II (juveniles) had a significantly greater capacity for LC-PUFA biosynthesis compared to stage I (fry to early juvenile). This enhanced capacity likely facilitates the conversion of C18 PUFA into LC-PUFA to support the metabolic demands of rapid growth.

LC-PUFAs are essential not only for individual growth but also for gonadal development and maturation, as they serve as precursors for the synthesis of sex hormones [26,27,28]. During stage III of this study, when gonadal development commenced, the levels of n-3 and n-6 LC-PUFAs in the liver and intestine were significantly higher than those at stage II. Similar findings have been reported in European perch (*Perca fluviatilis*), where the reproductive glands were found to accumulate LC-PUFA several months prior to spawning, in preparation for egg production [38]. These observations support the notion that fish exhibit an elevated requirement for EFAs as they approach the reproductive period [37]. The reduced proportions of n-3 and n-6 LC-PUFA in the muscle tissue of tilapia at stage III may indicate a physiological reallocation of these fatty acids from muscle to the developing gonads. Additionally, the expression levels of *fads2* and *elovl5* in both the liver and intestine were significantly upregulated at stage III compared to stages I and II, indicating an enhanced biosynthetic capacity for LC-PUFA to meet reproductive demand. In mammals, the expression of *Fads2* has been positively correlated with sex hormone levels [39]. Similarly, in broodstock species such as gilthead seabream (*Sparus aurata*), higher expression levels of key enzyme genes involved in LC-PUFA biosynthesis have been associated with better reproductive performance [1,40]. Collectively, these findings suggest that fish are capable of regulating LC-PUFA biosynthesis in a stage-specific manner to meet the physiological requirements of their gonads during maturation [41].

Interestingly, both the hepatic and intestinal LC-PUFA levels in tilapia remained elevated during the stage IV growth phase compared to stage II, yet they were still lower than the levels observed at stage III, particularly in the PT group. This trend was accompanied by a downregulation of intestinal *fads2* and *elovl5* expression at stage IV, indicating a reduced capacity for LC-PUFA biosynthesis in the later developmental stage. A similar pattern has been reported in European perch (*Perca fluviatilis*), where no significant difference in liver LC-PUFA content was observed between the pre-reproductive and reproductive stages. However, hepatic *fads2* expression was significantly higher in the earlier stage [38]. This may reflect early-stage LC-PUFA accumulation or a reduced physiological demand for LC-PUFA during the later stage, subsequently leading to downregulated biosynthesis. Notably, gonads exhibit higher levels of both n-3 and n-6 LC-PUFA compared to the gut, liver or muscle tissues, supporting the idea that LC-PUFA are preferentially accumulated in the gonads to fulfill reproductive functions [25,42,43]. Compared to the PO groups, fish in the PT groups had lower n-3 LC-PUFA but higher n-6 LC-PUFA levels in their gonads. Despite the reduced n-3 LC-PUFA content, gonadal development in the PT group was superior, suggesting that a diet rich in LA may be more beneficial for gonadal maturation in tilapia, particularly in males. Conversely, in species such as common carp (*Cyprinus carpio*) and yellow catfish (*Pelteobagrus fulvidraco*), higher dietary ALA ratios have been associated with improved gonadal development [25,43]. These discrepancies likely reflect interspecific differences in EFA requirements. For instance, tilapia have been reported to have a higher demand for n-6 fatty acids and demonstrate a strong capacity to utilize and preferentially incorporate LA during growth and development [44]. Collectively, these findings suggest that although the biosynthetic capacity for LC-PUFA may decline at the late growth stage (stage IV), tilapia continue to direct LC-PUFA toward gonadal tissues to support reproductive development. This highlights the physiological prioritization of LC-PUFA accumulation during gonad maturation and underscores the importance of tailoring dietary EFA profiles to species-specific requirements.

### 4.3. Comparison of the Biosynthetic Capacity of LC-PUFA in Male and Female Tilapia

Studies in mammals have shown that dietary EFAs can influence reproductive processes, and that sex hormones also modulate the biosynthesis of LC-PUFAs, resulting in gender-based differences in their synthetic capacity [45,46,47]. Further investigations have revealed that female mammals often possess a greater capacity to biosynthesize and deposit LC-PUFAs in comparison to their male counterparts [46,47].

Currently, there are no comprehensive reports specifically addressing sexual disparity in LC-PUFA biosynthetic capacity in fish. However, some studies have observed sex-based variations in tissue fatty acid composition. For instance, Wassef et al. investigated the fatty acid compositions of gilthead seabream (*Sparus aurata*) gonads and found that the n-6 LC-PUFA content was approximately 20% higher in testes than in ovaries, while no significant differences were observed for n-3 LC-PUFA [48]. Similarly, in the present study, n-6 LC-PUFA levels were approximately threefold higher in the testes compared to the ovaries, whereas n-3 LC-PUFA content showed no significant sex-based difference. In contrast, in amberjack (*Seriola fasciata*), both n-3 and n-6 LC-PUFA levels were significantly higher in the testes than in the ovaries [49]. Conversely, the Chinese tongue sole (*Cynoglossus semilaevis*) showed significantly lower levels of ARA in the testes than in the ovaries, while n-3 LC-PUFA levels were significantly higher in the testes [50]. These results collectively suggest that nutrient redistribution occurs during sexual maturation to meet differing physiological requirements, leading to changes in related metabolic processes [51]. This supports the possibility of sex-related differences in LC-PUFA biosynthesis in fish.

Furthermore, the present study revealed that the expression levels of key enzyme genes involved in LC-PUFA biosynthesis were significantly higher during the middle and late ontogenetic stages compared to the early stage, with a more pronounced upregulation observed in males than in females. The combined analysis of tissue fatty acid composition and gene expression patterns suggests that male tilapia may possess a greater capacity for LC-PUFA biosynthesis than their female counterparts.

## 5. Conclusions

In summary, this study provides novel insights into the biosynthesis of LC-PUFA in male and female tilapia across distinct ontogenetic stages (fry, juvenile, sub-adult and adult). The results reveal significant stage-dependent variations in LC-PUFA levels, their metabolic precursors and the expression levels of key enzymatic genes (*fads2* and *elovl*). Notably, compared to stage I (fry to early juvenile), tilapia exhibited a progressively enhanced ability to synthesize LC-PUFA from stages II to IV (juvenile to adult), with the peak biosynthetic capacity occurring at stage III (sub-adult), coinciding with the onset of sexual gland development. Male tilapia exhibited a relatively stronger biosynthetic capacity than females. Furthermore, diets rich in LA are found to promote growth and gonad maturation, particularly in males. These findings provide preliminary evidence of tissue-specific responses to dietary fatty acid profiles, particularly in the context of gene expression and morphological indicators. Long-term studies focusing on fecundity, egg quality, and larval performance are warranted to evaluate the functional significance of dietary lipid sources in aquaculture.

## Figures and Tables

**Table 1:** Ingredient, proximate composition and fatty acid composition of the experimental diets with different lipid sources.

Ingredient (%)	Dietary Groups	
	PO	PT
Fermented soybean meal	20	20
Cottonseed protein concentrate	10	10
Chicken powder	10	10
Meat and bone meal	6	6
Soybean protein concentrate	6	6
Spray-dried blood cells	4	4
Beer yeast meal	3	3
Pregelatinized starch	15	15
Wheat flour	6.5	6.5
Calcium dihydrogen phosphate	3	3
Choline chloride	2	2
Lecithin	2	2
Lysine	2	2
Taurine	1	1
*Bacillus subtilis*	1	1
Perilla oil ^1^	6	-
Peanut oil ^2^	-	6
Vitamin–mineral premix ^3^	2.5	2.5
Proximate composition (%)		
Dry matter	89.32	90.15
Ash	11.24	10.78
Crude protein	36.72	36.66
Crude lipid	7.56	7.23
Main fatty acids (% total fatty acids)		
16:0	16.78	13.33
18:0	5.48	4.66
SFA	19.12	22.91
16:1n-7	0.84	0.49
18:1n-9	24.05	38.12
MUFA	24.88	38.60
18:2n-6 (LA)	24.96	34.91
18:3n-3 (LNA)	20.32	2.21
PUFA	45.28	37.12

^1^ Perilla oil contained 12.84% LA and 64.31% ALA. ^2^ Peanut oil contained 31.37% LA and 1.18% ALA. ^3^ Consisting of the vitamin mixture and mineral compound. The amounts of the vitamin mixture and mineral compound were described in our previous study [16].

**Table 2:** qPCR primers sequence used in this study.

Genes	Primers	Sequence 5′—3′	Accession Number
Δ5Δ6 fads2	Δ5Δ6 fads2-F	CTGGTCATCGATCGAAAGGT	NM_001279623.1
	Δ5Δ6 fads2-R	GCGGCTTCAGAAACTTATGC	
Δ4 fads2	Δ4 fads2-F	CTTACTGTGCTCGGTGATT	XM_003440472.4
	Δ4 fads2-R	GGTCCTTGCTGAAGATGTT	
elovl5	elovl5-F	GGCTTCCTCCTCCGTCTAAA	NM_001279460.1
	elovl5-R	GTGCAAAGGTTGGTGGGTAG	
β-actin	β-actin-F	CAGGATGCAGAAGGAGATCACA	KJ126772.1
	β-actin-R	CGATCCAGACGGAGTATTTACG	

**Table 3:** Initial whole-body fatty acid composition of tilapia (% total fatty acids).

Main Fatty Acid	Groups	
	Male	Female
18:2n-6	7.27	8.93
20:3n-6	0.43	0.35
20:4n-6	2.56	2.14
18:3n-3	2.66	2.66
20:5n-3	2.40	2.62
22:5n-3	4.22	4.12
22:6n-3	16.01	14.84
SFA	33.31	32.40
MUFA	23.30	24.33
PUFA	35.04	35.54
n-3 LC-PUFA	23.07	22.00
n-6 LC-PUFA	3.69	3.62

Notes: values are Mean ± SE (*n* = 3).

**Table 4:** Fatty acid composition in the gonad of tilapia fed with experimental diets during the fourth growth stage (% total fatty acids).

	Dietary Groups	
	PO	PT
XY		
18:2n-6	7.65 ± 0.31 ^b^	12.39 ± 1.26 ^a^
20:4n-6	2.24 ± 0.26 ^b^	4.38 ± 0.07 ^a^
18:3n-3	4.39 ± 0.16 ^a^	2.66 ± 0.34 ^b^
20:5n-3	1.49 ± 0.21 ^a^	0.56 ± 0.09 ^b^
22:5n-3	2.13 ± 0.32 ^a^	0.68 ± 0.11 ^b^
22:6n-3	7.53 ± 0.39 ^a^	3.45 ± 0.91 ^b^
SFA	39.49 ± 1.44	38.29 ± 2.11
MUFA	14.27 ± 1.85 ^b^	20.10 ± 2.83 ^a^
PUFA	30.15 ± 1.58	28.79 ± 2.20
n-3 LC-PUFA	12.42 ± 0.48 ^a^	4.66 ± 0.96 ^b^
n-6 LC-PUFA	3.74 ± 0.15 ^b^	6.17 ± 0.07 ^a^
XX		
18:2n-6	10.56 ± 0.73 ^b^	16.17 ± 0.29 ^a^
20:4n-6	0.85 ± 0.04 ^b^	1.88 ± 0.03 ^a^
18:3n-3	12.04 ± 1.21 ^a^	2.02 ± 0.09 ^b^
20:5n-3	0.91 ± 0.08 ^a^	0.26 ± 0.04 ^b^
22:5n-3	1.44 ± 0.07 ^a^	0.48 ± 0.06 ^b^
22:6n-3	7.05 ± 0.12 ^a^	2.34 ± 0.04 ^b^
SFA	32.19 ± 0.90	30.35 ± 0.60
MUFA	24.89 ± 1.05 ^b^	32.95 ± 1.47 ^a^
PUFA	36.87 ± 2.21 ^a^	27.70 ± 0.53 ^b^
n-3 LC-PUFA	10.89 ± 1.56 ^a^	3.27 ± 0.49 ^b^
n-6 LC-PUFA	1.81 ± 0.32 ^b^	2.30 ± 0.18 ^a^

Values are means ± SE from three treatments (*n* = 3) with 9 fish per treatment. Different superscripts in the same rows indicate significant difference.

## Data Availability

Data is contained within the article or Supplementary Materials.

## Supplementary Materials

The following supporting information can be downloaded at https://www.mdpi.com/article/10.3390/life15081167/s1, Figure S1: Histological section analysis of the testes (A,B) and ovaries (C,D) of adult tilapia fed with diets PO (A,C) or PT (B,D) during the fourth growth period; Table S1:The final weight of tilapia during the four-growth stage (g); Table S2: Fatty acid compositions in the liver of tilapia among the four feeding stages (% total fatty acids); Table S3: Fatty acid compositions in the intestine of tilapia among the four feeding stages (% total fatty acids); Table S4: Fatty acid compositions in the muscle of tilapia among the four feeding stages (% total fatty acids).

## Abbreviations

The following abbreviations are used in this manuscript:

ALA	α-Linolenic acid
ARA	Arachidonic acid
DHA	Docosahexaenoic acid
EFA	Essential fatty acids
Elovl	Elongation of very long-chain fatty acids
EPA	Eicosapentaenoic acid
Fads	Fatty acid desaturase
LA	Linoleic acid
LC-PUFA	Long-chain polyunsaturated fatty acid
VO	Vegetable oil
