# Supplementary material for: Comparison of LC-PUFAs Biosynthetic Characteristics in Male and Female Tilapia at Different Ontogenetic Stages
Source: Life (Basel). 2025 Jul 23;15(8):1167. doi: 10.3390/life15081167 (PMC12387841; doi:10.3390/life15081167)
Supplement: Supplementary file 1 [file life-15-01167-s001.zip › life-3725895-supplementary.pdf]

Supplemental Figures and Tables

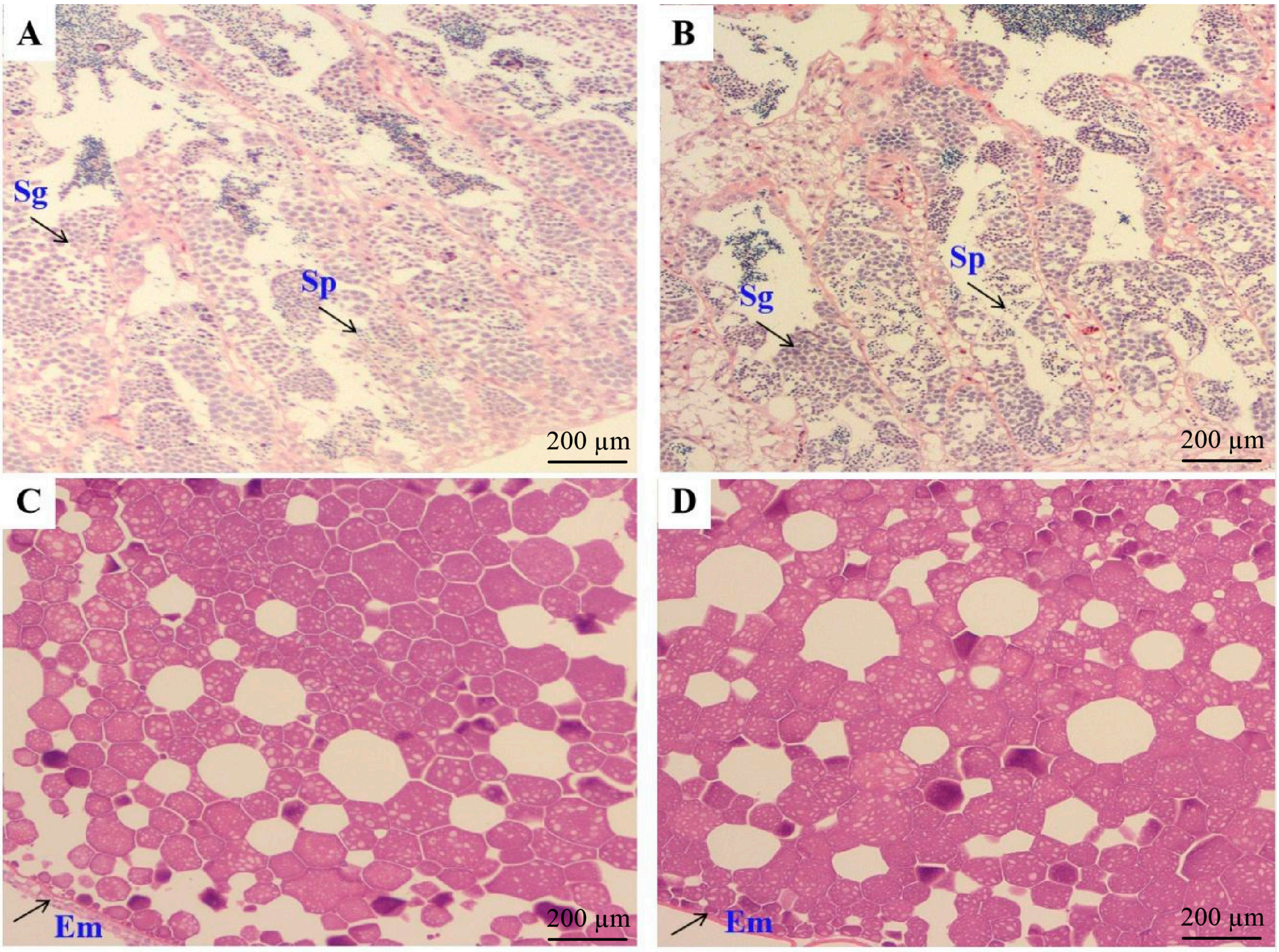

Figure S1. Histological section analysis of the testes (A, B) and ovaries (C, D) of adult tilapia fed with diets PO (A, C) or PT (B, D) during the fourth growth period

Table S1. The final weight of tilapia during the four-growth stage (g)

Growth stage	Male		Female	
	PO	PT	PO	PT
I	5.31±0.34	4.31±0.16	4.59±0.83	3.76±0.77
II	11.48±0.24	14.03±2.43	10.90±2.57	12.67±2.44
III	39.84±4.37	42.98±5.42	24.93±8.43	26.15±5.41
IV	88.63±5.43^{b}	159.74±13.74^{a}	52.52±2.15	61.07±5.65

Notes: Values are Mean ± SE (n = 3). Values in the same row of male or female group without sharing a common letter are significantly different ($p < 0.05$).

Table S2. Fatty acid compositions in the liver of tilapia among the four feeding stages (% total fatty acids)

	**Different feeding stages**			
	**I**	**II**	**III**	**IV**
**Male fish fed PO diets**				
18:2n-6	6.23±0.12^{b}	8.74±0.30^{a}	7.56±0.21^{a}	8.51±0.53^{a}
20:3n-6	0.53±0.02^{b}	0.78±0.01^{b}	1.06±0.04^{a}	0.81±0.03^{b}
20:4n-6	0.45±0.02^{a}	0.18±0.01^{b}	0.28±0.02^{a}	0.25±0.03^{ab}
18:3n-3	4.23±0.01^{b}	6.85±0.33^{a}	5.24±0.43^{a}	6.83±0.82^{a}
20:5n-3	1.02±0.01^{a}	0.61±0.07^{b}	0.61±0.04^{b}	0.71±0.03^{b}
22:5n-3	0.63±0.05^{b}	0.72±0.06^{ab}	0.83±0.07^{a}	1.31±0.13^{a}
22:6n-3	8.11±0.27^{a}	5.84±0.24^{b}	9.74±0.33^{a}	6.24±0.31^{ab}
SFA	40.71±3.27	38.84±2.38	43.01±2.95	38.31±1.26
MUFA	31.80±2.25	29.62±4.73	27.93±2.61	31.15±2.34
PUFA	22.73±1.29	28.05±1.15	26.85±2.24	27.61±1.47
n-3 LC-PUFA	10.23±0.47^{a}	9.84±0.15^{b}	11.21±0.63^{a}	10.30±0.28^{a}
n-6 LC-PUFA	1.63±0.08^{a}	1.03±0.03^{b}	1.54±0.06^{a}	1.17±0.07^{ab}
**Male fish fed PT diets**				
18:2n-6	8.51±0.21^{b}	11.33±0.17^{a}	10.44±0.41^{a}	11.04±0.15^{a}
20:3n-6	0.67±0.03^{b}	0.82±0.02^{b}	1.26±0.03^{a}	1.12±0.03^{ab}
20:4n-6	0.97±0.08^{a}	0.43±0.03^{b}	0.73±0.02^{a}	0.62±0.04^{ab}
18:3n-3	1.73±0.03^{b}	2.54±0.04^{a}	2.13±0.02^{a}	2.41±0.02^{a}
20:5n-3	0.84±0.03^{a}	0.21±0.02^{b}	0.33±0.02^{b}	0.24±0.01^{b}
22:5n-3	0.48±0.07^{b}	0.68±0.03^{a}	0.64±0.04^{b}	0.78±0.05^{a}
22:6n-3	3.34±0.12^{a}	1.62±0.18^{b}	4.03±0.31^{a}	1.96±0.20^{b}
SFA	40.51±2.61	39.06±2.18	41.72±3.91	36.07±1.68
MUFA	34.21±2.88	38.90±2.91	33.43±3.45	34.07±2.32
PUFA	19.11±0.21^{ab}	16.81±0.31^{b}	21.51±0.71^{a}	22.31±0.41^{a}
n-3 LC-PUFA	4.09±0.18^{a}	2.67±0.27^{b}	5.12±0.33^{a}	3.71±0.34^{ab}
n-6 LC-PUFA	2.31±0.07^{a}	1.51±0.06^{b}	2.15±0.05^{a}	1.98±0.04^{ab}
**Female fish fed PO diets**				
18:2n-6	6.87±0.13^{b}	9.33±0.22^{a}	7.63±0.41^{a}	8.42±0.50^{a}
20:3n-6	0.61±0.02^{b}	0.67±0.03^{b}	0.94±0.02^{a}	0.78±0.02^{a}

20:4n-6	0.43±0.05^{a}	0.17±0.01^{b}	0.29±0.03^{a}	0.26±0.01^{ab}
18:3n-3	3.44±0.04^{b}	8.33±0.37^{a}	6.62±0.03^{a}	7.42±0.52^{a}
20:5n-3	1.02±0.04^{a}	0.51±0.04^{b}	0.61±0.05^{b}	0.61±0.06^{b}
22:5n-3	0.41±0.08^{b}	1.01±0.05^{a}	0.96±0.06^{a}	1.04±0.07^{a}
22:6n-3	8.73±0.34^{a}	6.75±0.44^{b}	9.71±0.72^{a}	8.61±0.83^{a}
SFA	41.20±3.12	35.82±2.68	42.34±1.87	37.15±1.68
MUFA	31.74±1.81	33.52±2.62	27.21±3.87	26.34±2.37
PUFA	21.88±1.37	26.51±1.37	26.83±1.56	31.80±2.54
n-3 LC-PUFA	10.82±0.31^{a}	8.94±0.62^{b}	11.47±0.92^{a}	10.71±1.43^{a}
n-6 LC-PUFA	1.52±0.04^{a}	1.01±0.11^{b}	1.42±0.08^{a}	1.33±0.02^{ab}
**Female fish fed PT diets**				
18:2n-6	8.93±0.42^{b}	11.17±0.53^{a}	11.32±0.60^{a}	12.61±0.70^{a}
20:3n-6	0.75±0.02^{b}	0.83±0.01^{ab}	1.26±0.02^{a}	1.11±0.02^{a}
20:4n-6	1.33±0.01^{a}	0.42±0.02^{c}	0.88±0.03^{b}	0.62±0.07^{b}
18:3n-3	1.67±0.01^{b}	2.62±0.21^{a}	2.44±0.14^{a}	2.57±0.13^{a}
20:5n-3	0.41±0.01^{a}	0.20±0.01^{b}	0.28±0.01^{b}	0.21±0.01^{b}
22:5n-3	0.34±0.03^{b}	0.61±0.03^{a}	0.51±0.02^{a}	0.57±0.02^{a}
22:6n-3	1.86±0.05^{a}	1.23±0.03^{b}	2.17±0.31^{a}	1.51±0.11^{b}
SFA	41.92±2.51	36.21±1.87	38.01±3.71	36.81±2.54
MUFA	36.61±2.11	42.31±3.01	35.41±2.38	37.81±2.73
PUFA	15.51±2.41^{b}	15.11±1.33^{b}	20.81±2.74^{a}	23.21±2.54^{a}
n-3 LC-PUFA	2.91±0.03^{ab}	2.44±0.02^{b}	3.05±0.06^{a}	2.67±0.11^{ab}
n-6 LC-PUFA	2.28±0.23^{a}	1.58±0.07^{b}	2.23±0.04^{a}	1.92±0.12^{ab}

Notes: Values are Mean ± SE (n = 3). Values in the same row of fish among different growth stages without sharing a common letter are significantly different ($p < 0.05$).

Table S3. Fatty acid compositions in the intestine of tilapia among the four feeding stages (% total fatty acids)

	Different feeding stages			
	I	II	III	IV
**Male fish fed PO diets**				
18:2n-6	7.02±0.07^{b}	11.13±0.52^{a}	10.23±1.64^{a}	11.32±0.52^{a}
20:3n-6	0.48±0.02^{b}	0.55±0.01^{b}	1.19±0.03^{a}	0.91±0.02^{a}
20:4n-6	0.36±0.01^{a}	0.28±0.02^{b}	0.31±0.03^{ab}	0.33±0.02^{ab}
18:3n-3	3.01±0.12^{c}	11.21±0.63^{a}	6.01±0.30^{b}	7.91±0.43^{b}
20:5n-3	0.43±0.02^{b}	0.35±0.04^{b}	0.53±0.02^{a}	0.33±0.02^{b}
22:5n-3	0.47±0.09^{b}	0.62±0.12^{b}	1.33±0.14^{a}	1.52±0.12^{a}
22:6n-3	4.42±0.33^{b}	2.66±0.13^{b}	9.85±1.40^{a}	7.16±0.45^{a}
SFA	42.21±3.47^{a}	34.31±2.72^{b}	40.61±2.34^{a}	37.71±3.24^{ab}
MUFA	31.81±2.34^{a}	32.71±3.44^{a}	24.71±2.61^{b}	26.21±2.72^{ab}
PUFA	24.01±0.61^{b}	27.01±0.51^{b}	32.91±0.71^{a}	31.21±0.51^{a}
n-3 LC-PUFA	5.91±0.32^{b}	4.11±0.75^{b}	12.31±0.23^{a}	10.64±0.66^{a}
n-6 LC-PUFA	1.36±0.03^{b}	1.07±0.02^{b}	1.84±0.06^{a}	1.72±0.03^{a}
**Male fish fed PT diets**				
18:2n-6	8.12±0.12^{b}	12.83±0.93^{a}	10.66±0.33^{a}	16.24±0.62^{a}

20:3n-6	0.77±0.03^{b}	0.85±0.02^{b}	1.61±0.03^{a}	1.43±0.02^{a}
20:4n-6	0.56±0.06^{a}	0.31±0.01^{b}	0.38±0.03^{ab}	0.41±0.02^{a}
18:3n-3	2.11±0.32^{b}	3.71±0.22^{a}	3.03±0.13^{a}	3.42±0.12^{a}
20:5n-3	0.35±0.04^{b}	0.31±0.03^{b}	0.44±0.03^{a}	0.30±0.04^{b}
22:5n-3	0.51±0.11^{b}	0.41±0.01^{b}	0.71±0.01^{a}	1.01±0.21^{a}
22:6n-3	2.23±0.01^{b}	1.52±0.11^{b}	3.84±0.41^{a}	2.56±0.11^{b}
SFA	38.10±2.17^{a}	32.93±2.42^{b}	38.04±1.71^{a}	36.90±2.62^{ab}
MUFA	33.74±2.45^{a}	38.13±3.86^{a}	25.85±1.42^{b}	29.43±1.83^{ab}
PUFA	22.61±2.12^{b}	24.93±1.91^{b}	27.76±1.52^{a}	30.94±2.83^{a}
n-3 LC-PUFA	4.12±0.16^{a}	2.78±0.12^{b}	5.32±0.51^{a}	4.98±0.13^{a}
n-6 LC-PUFA	1.72±0.05^{ab}	1.51±0.03^{b}	2.31±0.06^{a}	2.18±0.05^{a}
**Female fish fed PO diets**				
18:2n-6	7.06±0.13^{b}	13.42±0.47^{a}	10.83±0.22^{a}	12.24±0.63^{a}
20:3n-6	0.54±0.03^{b}	0.61±0.02^{b}	1.09±0.03^{a}	0.85±0.03^{a}
20:4n-6	0.35±0.04^{a}	0.16±0.04^{b}	0.26±0.02^{b}	0.22±0.03^{b}
18:3n-3	5.23±0.30^{c}	13.53±1.52^{a}	8.25±0.44^{b}	9.27±0.62^{ab}
20:5n-3	0.44±0.02^{b}	0.69±0.03^{b}	0.63±0.01^{a}	0.82±0.08^{a}
22:5n-3	0.51±0.01^{b}	0.83±0.03^{a}	0.78±0.06^{a}	0.81±0.08^{a}
22:6n-3	4.41±0.31^{b}	3.07±0.41^{b}	7.09±0.81^{a}	5.64±0.41^{ab}
SFA	38.23±1.44^{a}	32.64±1.91^{b}	39.75±1.55^{a}	35.68±3.08^{ab}
MUFA	34.33±1.57^{a}	31.53±2.47^{a}	24.28±2.18^{b}	25.33±1.33^{b}
PUFA	23.52±2.71^{b}	30.11±321^{a}	33.24±2.21^{a}	34.82±2.67^{a}
n-3 LC-PUFA	6.91±0.31^{b}	5.11±0.22^{b}	10.46±0.61^{a}	8.89±0.57^{a}
n-6 LC-PUFA	1.12±0.04^{b}	0.97±0.06^{b}	1.55±0.11^{a}	1.43±0.12^{a}
**Female fish fed PT diets**				
18:2n-6	8.07±0.23^{b}	17.46±0.51^{a}	15.28±0.34^{a}	17.35±1.09^{a}
20:3n-6	0.88±0.23^{b}	1.00±0.23^{b}	1.50±0.05^{a}	1.52±0.11^{b}
20:4n-6	0.42±0.07^{a}	0.34±0.12^{b}	0.36±0.04^{b}	0.37±0.06^{b}
18:3n-3	2.18±0.08^{b}	3.12±0.17^{a}	3.53±0.14^{a}	3.36±0.20^{a}
20:5n-3	0.33±0.06^{b}	0.46±0.02^{a}	0.32±0.04^{b}	0.56±0.07^{a}
22:5n-3	0.37±0.02^{b}	0.42±0.02^{b}	0.39±0.04^{b}	0.47±0.05^{a}
22:6n-3	2.31±0.08^{a}	1.49±0.03^{b}	3.43±0.05^{a}	2.71±0.19^{a}
SFA	39.81±2.29	31.81±1.72	34.41±2.27	38.01±2.76
MUFA	37.21±2.87	36.91±3.37	31.21±3.58	30.01±3.44
PUFA	20.21±2.18^{b}	25.31±1.99^{ab}	27.21±1.74^{a}	27.01±2.56^{a}
n-3 LC-PUFA	3.81±0.03^{b}	2.37±0.31^{b}	5.33±0.21^{a}	4.84±0.02^{a}
n-6 LC-PUFA	1.54±0.23^{b}	1.52±0.04^{b}	2.13±0.02^{a}	2.08±0.21^{a}

Notes: Values are Mean ± SE (n = 3). Values in the same row of fish among different growth stages without sharing a common letter are significantly different (p < 0.05).

Table S4. Fatty acid compositions in the muscle of tilapia among the four feeding stages (% total fatty acids)

	Different feeding stages

	I	II	III	IV
**Male fish fed PO diets**				
18:2n-6	8.44±0.22[b]	11.21±0.21[a]	11.92±0.60[a]	10.57±0.17[a]
20:3n-6	0.60±0.02[a]	0.48±0.02[b]	0.44±0.04[b]	0.49±0.22[a]
20:4n-6	0.62±0.03[a]	0.44±0.08[b]	0.38±0.04[b]	0.43±0.02[b]
18:3n-3	2.83±0.01[c]	8.53±0.53[b]	10.81±0.51[a]	7.04±0.61[b]
20:5n-3	0.51±0.04[a]	0.28±0.04[b]	0.44±0.01[a]	0.29±0.09[b]
22:5n-3	1.28±0.15	1.31±0.01	1.02±0.07	1.15±0.11
22:6n-3	7.17±0.27[a]	6.45±0.18[b]	4.76±0.31[b]	6.71±0.71[a]
SFA	35.60±0.61	33.25±0.72	35.42±1.13	32.80±0.92
MUFA	27.42±0.82	30.50±0.42	23.82±0.51	26.53±1.62
PUFA	29.66±2.92	30.74±1.91	31.75±1.32	34.52±2.04
n-3 LC-PUFA	9.88±0.48[a]	8.52±1.14[a]	6.94±0.53[b]	9.31±0.81[a]
n-6 LC-PUFA	1.69±0.16[a]	1.53±0.04[ab]	1.33±0.01[b]	1.46±0.06[ab]
**Male fish fed PT diets**				
18:2n-6	9.47±1.21[b]	14.64±0.32[a]	16.22±0.13[a]	15.83±0.31[a]
20:3n-6	0.87±0.04	0.63±0.02	0.55±0.03	0.67±0.02
20:4n-6	0.72±0.15[a]	0.56±0.12[b]	0.41±0.01[b]	0.56±0.01[b]
18:3n-3	2.32±0.21[b]	3.41±0.13[a]	3.80±0.30[a]	3.51±0.16[a]
20:5n-3	0.64±0.01[a]	0.33±0.01[b]	0.62±0.11[a]	0.36±0.01[b]
22:5n-3	0.81±0.01[a]	0.37±0.11[b]	0.51±0.01[a]	0.48±0.01[ab]
22:6n-3	3.01±0.11[a]	2.41±0.11[ab]	2.11±0.11[b]	2.61±0.11[ab]
SFA	36.21±0.28	30.31±0.85	35.11±0.35	31.61±0.84
MUFA	30.81±1.13	31.18±0.54	30.81±0.92	30.81±1.65
PUFA	29.54±0.65	26.75±0.21	27.66±0.45	33.33±2.05
n-3 LC-PUFA	4.92±0.24[a]	3.73±0.13[a]	2.81±0.21[b]	3.91±0.23[a]
n-6 LC-PUFA	2.21±0.21[a]	1.71±0.02[b]	1.65±0.03[b]	1.82±0.05[b]
**Female fish fed PO diets**				
18:2n-6	8.22±0.06[b]	12.64±0.37[a]	13.67±0.40[a]	11.63±0.24[a]
20:3n-6	0.69±0.02[a]	0.36±0.03[b]	0.34±0.01[b]	0.41±0.03[b]
20:4n-6	0.77±0.01[a]	0.46±0.01[b]	0.42±0.02[b]	0.56±0.01[b]
18:3n-3	2.95±0.11[b]	10.44±0.45[a]	10.42±0.64[a]	9.11±0.76[a]
20:5n-3	0.83±0.03[a]	0.57±0.12[b]	0.53±0.02[b]	0.54±0.05[b]
22:5n-3	1.42±0.08[a]	1.21±0.14[a]	0.78±0.06[b]	1.10±0.13[ab]
22:6n-3	6.14±0.22[a]	5.44±0.44[a]	4.01±0.02[b]	5.81±0.57[a]
SFA	35.51±1.55	33.61±0.25	35.01±0.74	31.81±0.77
MUFA	30.47±2.14[a]	28.18±0.81[ab]	25.68±0.61[b]	27.44±1.61[ab]
PUFA	27.55±1.41[b]	31.44±0.61[ab]	35.85±1.21[a]	35.17±1.61[a]
n-3 LC-PUFA	8.87±0.22[a]	8.03±0.65[a]	6.57±0.16[b]	8.05±0.82[a]
n-6 LC-PUFA	1.69±0.13[a]	1.22±0.02[b]	1.04±0.01[b]	1.34±0.04[b]
**Female fish fed PT diets**				
18:2n-6	10.72±1.34[b]	16.42±1.25[a]	16.05±1.35[a]	17.41±1.39[a]
20:3n-6	0.83±0.02[a]	0.64±0.04[b]	0.57±0.03[b]	0.60±0.04[b]

20:4n-6	0.83±0.06[a]	0.53±0.03[b]	0.47±0.03[b]	0.59±0.05[b]
18:3n-3	2.43±0.12[b]	3.70±0.21[a]	3.73±0.20[a]	3.75±0.14[a]
20:5n-3	0.59±0.04[a]	0.27±0.04[b]	0.47±0.12[a]	0.25±0.02[b]
22:5n-3	0.81±0.07[a]	0.41±0.01[b]	0.37±0.01[b]	0.36±0.02[b]
22:6n-3	3.01±0.13[a]	2.61±0.11[b]	1.71±0.03[b]	2.71±0.04[a]
SFA	37.71±2.81[a]	32.51±3.62[ab]	35.21±2.32[a]	29.71±1.27[b]
MUFA	28.31±1.24	30.21±2.08	30.01±3.44	32.31±2.58
PUFA	25.31±0.91[b]	29.11±0.71[ab]	31.51±0.71[a]	33.61±0.31[a]
n-3 LC-PUFA	4.41±0.23[a]	3.51±0.06[ab]	2.63±0.03[b]	3.81±0.18[a]
n-6 LC-PUFA	2.51±0.11[a]	1.67±0.13[b]	1.51±0.07[b]	1.73±0.03[b]

Notes: Values are Mean ± SE (n = 3). Values in the same row of fish among different growth stages without sharing a common letter are significantly different ($p < 0.05$).
